# Predicting Iron Deficiencies Using Routine Complete Blood Cell Count Parameters: A Machine Learning Approach and Evaluation

**DOI:** 10.3390/jcm15124783

**Published:** 2026-06-19

**Authors:** Davide Negrini, Laura Pighi, Simone Mignolli, Gian Luca Salvagno, Giuseppe Lippi

**Affiliations:** Section of Clinical Biochemistry, University of Verona, 37134 Verona, Italy; davide.negrini@aovr.veneto.it (D.N.); laura.pighi2@aovr.veneto.it (L.P.); simone.mignolli_02@studenti.univr.it (S.M.); gianluca.salvagno@univr.it (G.L.S.)

**Keywords:** machine learning, prediction algorithms, blood cell count, iron deficiencies

## Abstract

**Background/Objectives:** Iron deficiency remains a prevalent condition, needing specific laboratory tests for diagnosis. This study aimed to evaluate whether routine complete blood cell count (CBC) parameters can be used within a machine learning framework to predict low ferritin and low transferrin saturation, used as biochemical markers of altered iron status, potentially supporting more targeted laboratory test utilization. **Methods:** In this single-center retrospective outpatient study, we analyzed 32,437 records from subjects undergoing both complete blood cell count and iron metabolism testing between 2023 and 2026. Low ferritin and low transferrin saturation were defined using sex-specific thresholds. Low ferritin was present in 14,344 subjects (44.2%), whereas low transferrin saturation was present in 7791 subjects (24.0%). After cleaning data and excluding incomplete records, demographic variables and CBC indices were tested as potential predictors. The dataset was split into training and test sets with stratified sampling. Multiple supervised machine learning models, including logistic regression, decision tree, random forest, XGBoost, support vector machine, k-nearest neighbors, and Naive Bayes, were trained. Hyperparameter tuning and model selection were performed using repeated stratified 10-fold cross-validation, optimizing the area under the curve (AUC). Model performance was assessed by AUC, sensitivity, and specificity, and validated on an independent test set. **Results:** All models showed predictive capability for low ferritin and low transferrin saturation using CBC parameters alone. Ensemble methods, especially random forest and XGBoost, reached the best performance (AUC values of 0.80–0.87 for ferritin and 0.85–0.96 for transferrin saturation). Sensitivity and specificity were balanced, supporting clinical screening applicability. Results were maintained across validation and confirmed in the test set. Prediction of transferrin saturation showed slightly higher accuracy than ferritin. Feature importance analysis identified mean corpuscular volume (MCV), mean corpuscular hemoglobin (MCH), and red blood cell distribution width (RDW) as key predictors. **Conclusions:** CBC-based machine learning models may help identify subjects with low ferritin or low transferrin saturation, supporting subsequent targeted assessment of iron status.

## 1. Introduction

Anemia is a significant public health problem worldwide, affecting both high- and low-income countries, and is characterized by decreased hemoglobin (HB) levels and a reduced number of circulating red blood cells (RBCs), resulting in an insufficient erythrocyte mass to meet physiological demands. Laboratory findings typically include decreased HB, hematocrit (HT), and RBC count. Rather than a disease in itself, anemia represents a manifestation of an underlying disorder. In this context, iron deficiency represents one of the most relevant underlying conditions and remains among the most common nutritional deficiencies in Europe, affecting a substantial proportion of the population [1]. Older adults are at increased risk of iron deficiency, as aging is frequently associated with inadequate dietary intake, reduced gastrointestinal absorption often related to proton pump inhibitor use, and chronic blood loss due to angiodysplasia, gastrointestinal malignancies, or concomitant antithrombotic therapy [2].

Laboratory investigations for diagnosing iron deficiency and anemia include a variety of tests, such as complete blood count (CBC), serum iron, ferritin, transferrin, and transferrin saturation. The diagnostic workup of anemia should follow a stepwise approach in which the CBC serves as the initial investigation and guides further laboratory testing, whereas iron studies, including ferritin and transferrin parameters, are performed only when anemia or microcytosis is detected, to ensure appropriate and cost-effective use of laboratory resources [3,4].

CBC parameters are inexpensive, widely available, and routinely used in clinical practice. Importantly, erythrocyte indices such as mean corpuscular volume (MCV), mean corpuscular hemoglobin (MCH), and RBC distribution width (RDW) reflect alterations in erythropoiesis and may hence provide indirect evidence of iron-restricted erythropoiesis even before biochemical abnormalities become evident.

Machine learning methods have been applied in laboratory medicine to analyze laboratory data and to support diagnostic decision-making [5]. The large volume of laboratory data generated in clinical practice has allowed the development of models for earlier detection and more efficient use of laboratory resources. Previous studies have shown that machine learning can be integrated into laboratory medicine to support diagnostics and improve test utilization. Laboratory data are highly standardized and quantitative, which makes them suitable for machine learning applications. These approaches can improve test interpretation, support risk stratification, and contribute to more appropriate use of laboratory tests [6]. The value of this approach has been demonstrated in different diagnostic settings, where machine learning models based on routinely available laboratory and biochemical parameters have shown good performance in disease detection and classification [7,8]. The same analytical approach has also been applied to routine laboratory data to identify conditions that may not be easily detected by conventional procedures, including laboratory errors and preanalytical interferences such as wrong-blood-in-tube events and contamination from intravenous fluids [9,10].

These studies clearly show that laboratory data obtained in clinical practice can reveal patterns that are hardly detectable with conventional analytical approaches. Based on these considerations, the aim of our study was to evaluate the feasibility of a machine learning approach using only CBC parameters to identify subjects at risk of iron deficiency.

## 2. Materials and Methods

### 2.1. Dataset and Targeted Markers

We extracted anonymized data from the local Laboratory Information System (LIS) of all outpatients referred to our Laboratory who had complete blood count (CBC), serum iron, ferritin, and transferrin requested from the same blood collection between 1 January 2023 and 31 January 2026. Because inclusion required the simultaneous availability of CBC and iron metabolism tests, the study population represents a selected outpatient testing population rather than a general population undergoing CBC alone.

Outpatients were included if they were aged 10–99 years and had CBC, serum iron, ferritin, and transferrin measured from the same blood collection. Subjects with missing or incomplete socioeconomic or laboratory data, age outside the predefined range, or absence of paired CBC and iron metabolism testing from the same blood collection were excluded. No imputation was hence needed, and only patients aged 10–99 (inclusive) were kept. The age range was selected to obtain a broad outpatient laboratory cohort rather than a group limited to patients with an established diagnosis of iron deficiency anemia. This approach was considered appropriate for a machine-learning model aimed to evaluate whether routine CBC parameters can identify biochemical evidence of iron deficiency in daily laboratory practice.

CBC tests were performed using a Sysmex XN hematological system (Sysmex Corp., Kobe, Japan), while iron, ferritin, and transferrin in plasma were measured on Cobas c701 with proprietary test kits (Roche Diagnostics AG, Risch-Rotkreuz, Switzerland).

The final dataset was completely anonymized and included only the following columns: age (years), sex (Male or Female), RBC (×10^12^/L) count, HB (g/L), HT (L/L), MCV (fL), MHC (pg), mean corpuscular hemoglobin concentration (MCHC, g/L), RDW (%), plasma iron concentration (µmol/L), plasma ferritin concentration (µg/L), plasma transferrin concentration (g/L). Transferrin saturation in % was calculated using the following formula [(iron × 5.5845)/(transferrin × 138.9)] [11,12].

Targeted biomarkers for iron deficiency identification were (A) low ferritin concentration, considered as lower than 30 µg/L in females and 50 µg/L in males, and (B) low transferrin saturation, considered as lower than 15% in females and 20% in males.

These thresholds were used as biochemical criteria to define low ferritin and low transferrin saturation in the present analysis. Ferritin concentrations below 30 µg/L have been reported as indicative of reduced iron stores, whereas higher decision limits, about 45–50 µg/L, have been proposed to increase diagnostic sensitivity, especially in settings in which ferritin may be influenced by inflammation [13,14]. Low transferrin saturation was used as an index of reduced circulating iron availability; values below about 15–16% have been associated with iron-restricted erythropoiesis [15], while a threshold below 20% is used in clinical guidelines and reviews [16]. For this reason, the two targets were interpreted as biochemical classifications of iron status rather than as definitive clinical diagnoses of iron deficiency anemia.

### 2.2. Pipeline Implementation

A supervised machine learning approach was adopted to develop and compare binary classification models for predicting two distinct iron-deficiency biomarkers using routine CBC parameters alone. Two separate but methodologically identical analysis pipelines were implemented, one for each target biomarker.

For each pipeline, only the parameters age, sex, RBC, HB, HT, MCV, MCH, MCHC, and RDW were given as input, removing the values of the iron metabolism biomarkers, and including only a binary label as the prediction target (presence or absence of the low value).

To implement the desired pipeline, we created two R scripts (R Core Team (2025). R: A Language and Environment for Statistical Computing. R Foundation for Statistical Computing, Vienna, Austria) differing only in the biomarker targeted, written in Visual Studio Code 1.119 (Microsoft Corp., Redmond, WA, USA) and reviewed for optimization using GitHub Copilot with Claude Opus 4.6 (Anthropic PBC, San Francisco, CA, USA).

The R script was then run on R 4.5.2 (31 October 2025) with RStudio Pro 2025.09.1. (Posit Software PBC, Boston, MA, USA) on a x64-pc-linux-gnu platform.

First of all, the dataset was split into a training set (70%) and a held-out test set (30%) using stratified random sampling via the createDataPartition function of “caret” package [17], which preserves the original class distribution in both subsets. A constant random seed was used for all analyses to ensure comparable splits.

Most models (all except XGBoost and Naive Bayes) were trained and tuned using a unified framework via the “caret” package, while the independent test set was used only for final performance assessment. A repeated stratified 10-fold cross-validation scheme (3 repeats, 30 total resampling iterations) was applied on the training set. The optimization metric was the area under the receiver operating characteristics (ROC) curve (AUC). Cross-validated estimates of AUC, sensitivity, and specificity (mean ± standard deviation across folds) were recorded for all models using the twoClassSummary function. Class probability estimation was enabled for all classifiers to support ROC analysis.

### 2.3. Classifiers Evaluation

Nine classifiers spanning different algorithmic families were trained and evaluated:

(a) Logistic Regression (caret method “glm”, predictions obtained by applying a 0.5 probability threshold to the predicted probabilities).
(b) Basic Decision Tree (caret method “rpart”, fixed complexity parameter cp = 0.01).
(c) Optimized Decision Tree (caret method “rpart”, with hyperparameter tuning of the complexity parameter (cp) over a grid ranging from 0.001 to 0.05 in steps of 0.005. The optimal cp was selected as the value maximizing the cross-validated AUC).
(d) Basic Random Forest (caret method “rf”, with 500 trees and fixed mtry = 5).
(e) Tuned Random Forest: (caret method “rf”, with 500 trees with hyperparameter tuning of mtry (values: 2, 3, 4, 5, 6) to maximize cross-validated AUC).
(f) XGBoost (Extreme Gradient Boosting) (trained using “xgboost” package, with a custom repeated cross-validation procedure (10-fold × 3 repeats) implemented using createMultiFolds, ensuring methodological consistency with the other models; grid search was performed over the following hyperparameters: number of boosting rounds (nrounds: 50, 100, 150), maximum tree depth (max_depth: 3, 6), learning rate (eta: 0.1, 0.3)).
(g) Support Vector Machine (SVM) with a radial basis function (caret method “svmRadial”, trained with automatic tuning of the sigma and cost (C) parameters with tuneLength = 5; features were centered and scaled as part of the caret preprocessing pipeline).
(h) k-Nearest Neighbors (k-NN): A k-NN classifier (caret method “knn”, trained with hyperparameter tuning of k over odd values from 3 to 25, optimal k selected by maximizing cross-validated AUC on the training set; features were centered and scaled as part of the caret preprocessing pipeline).
(i) Naive Bayes, assuming Gaussian feature distributions (trained using the “e1071” package, with a custom repeated cross-validation procedure (10-fold × 3 repeats), was implemented using createMultiFolds, ensuring methodological consistency with the other models).

Each model was evaluated on the held-out test set (30% of data, never used during training or tuning). The following metrics were computed from the confusion matrix with the positive class set to “1”: Accuracy, Balanced Accuracy (average of sensitivity and specificity), Sensitivity, Specificity, Positive Predictive Value, Negative Predictive Value, F1-score (harmonic mean of precision and sensitivity).

For all models providing class probability estimates, ROC curves were constructed on the test set, and the AUC was computed using the “pROC” package [18]. The 95% confidence interval for each AUC was estimated using the DeLong method [19].

To determine whether the discriminative performance of the best-performing model was statistically significantly different from each of the other models, pairwise DeLong tests (roc.test function, “pROC” package) were performed on the test-set ROC curves. Statistical significance was assessed at alpha = 0.05. The best model was identified as the one with the highest test-set AUC and served as the reference for all pairwise comparisons. Moreover, although the current models are not yet directly applicable in current clinical practice, the Brier score was calculated to evaluate model calibrations.

## 3. Results

The final dataset had a total of 32,437 rows with complete data about the 9 prediction factors (age, sex, RBC, HB, HT, MCV, MCH, MCHC, RDW) and the selected biomarkers, of which 15,917 for males and 16,520 for females; age range 10–99 years, median age 65 (25–75° percentile: 48–78). With a 70:30 split, we had 22,707 rows in the training set and 9730 in the test set.

**Figure 1:**
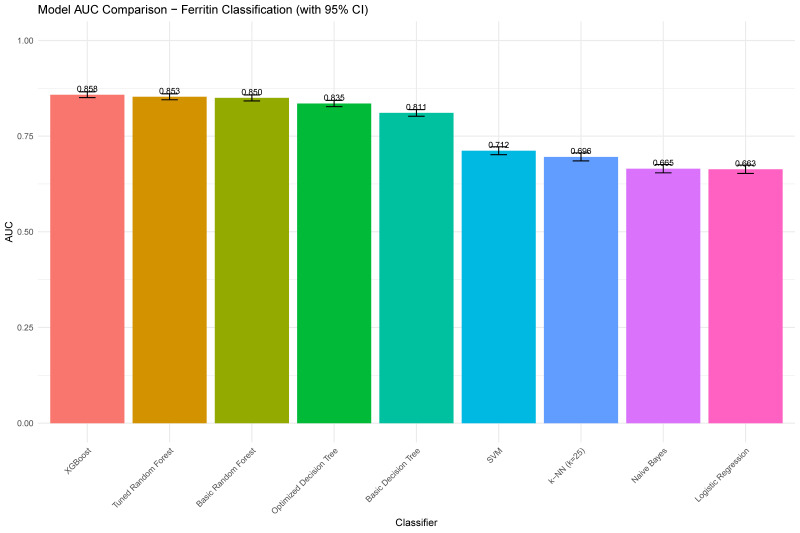
Graphical comparison of AUCs of the different classifiers for the low ferritin target. AUC, area under the curve.

For the low ferritin, we had a target distribution of 18,093 negative (non-low value, 55.8%) and 14,344 positive (low value, 44.2%) cases; results of the different classifiers on the holdout test set are reported in Table 1. In Figure 1 a graphical AUC comparison is reported.

**Figure 2:**
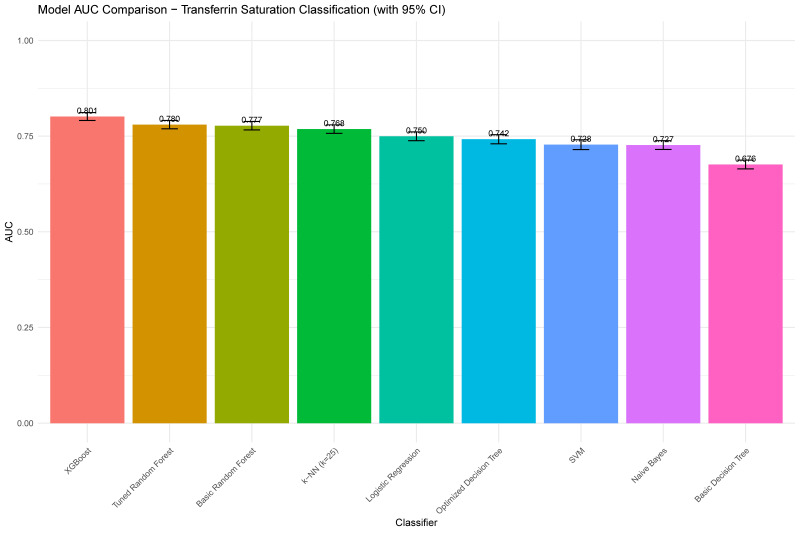
Graphical comparison of AUCs of the different classifiers for the low transferrin saturation target. AUC, area under the curve.

Given the low transferrin saturation, we set a target distribution of 24,646 negative (non-low value, 76.0%) and 7791 positive (low value, 24.0%) cases. Results of the different classifiers on the holdout test set are reported in Table 2. In Figure 2 a graphical AUC comparison is reported.

**Figure 3:**
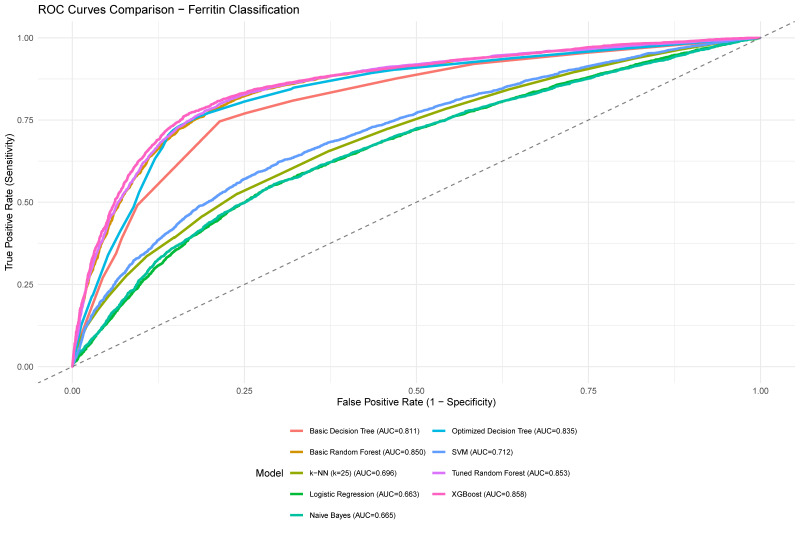
ROC curves of the different classifiers for the low ferritin target. ROC, Receiver Operating Characteristics.

**Figure 4:**
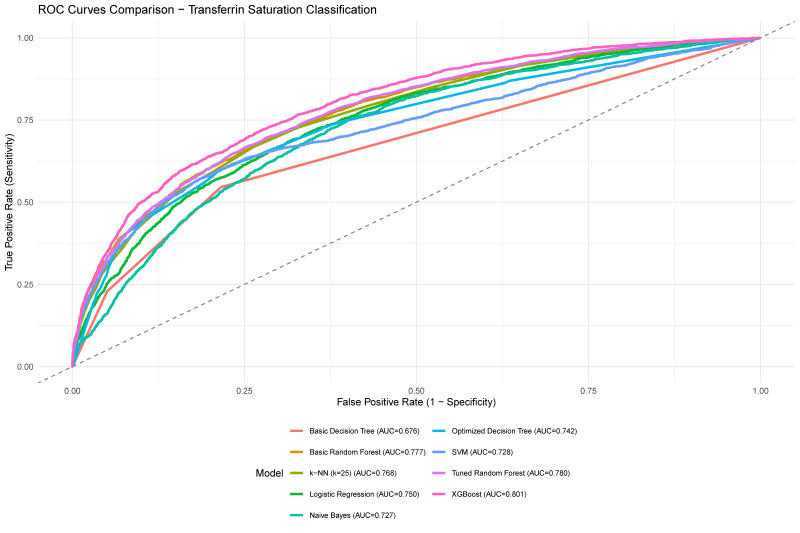
ROC curves of the different classifiers for the low transferrin saturation target. ROC, Receiver Operating Characteristics.

For both targets, XGBoost achieved the highest AUC, and the model, compared with all others evaluated using the DeLong test, always had a *p*-value < 0.05; ROC curves are displayed in Figure 3 and Figure 4.

**Figure 5:**
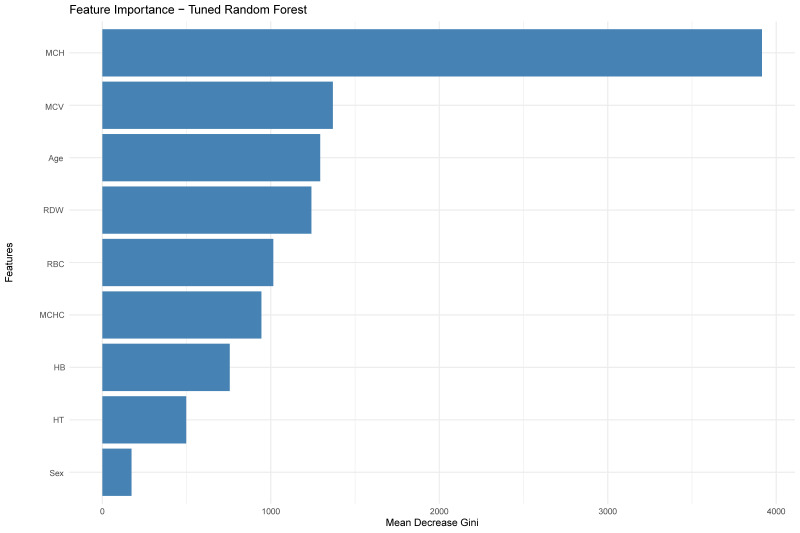
Feature importance extracted from the tuned Random Forest model using the Mean Decrease Gini index for the low ferritin target.

**Figure 6:**
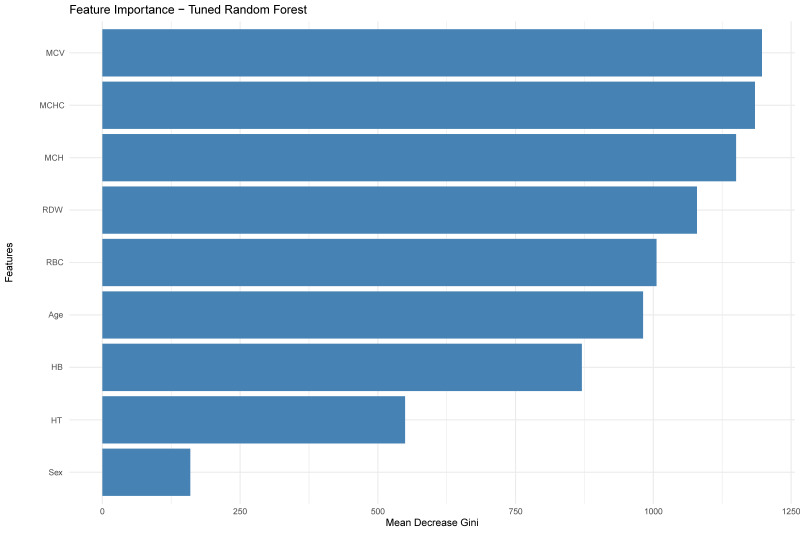
Feature importance extracted from the tuned Random Forest model using the Mean Decrease Gini index for the low transferrin saturation target.

As an additional evaluation, feature importance was extracted from the tuned Random Forest model (although it may not be the best-performing model, it is favored due to the greater ease of data retrieval) using the Mean Decrease Gini index, which quantifies the total decrease in node impurity (Gini impurity) attributable to each feature across all trees in the ensemble. Features were ranked in descending order of importance and are presented graphically for the two targets in Figure 5 and Figure 6. For the ferritin and transferrin saturation targets, the most important predictors were MCH and MCV, respectively.

## 4. Discussion

Machine learning methods have recently been used in laboratory medicine for analyzing complex quantitative datasets. Supervised models are trained on predefined laboratory variables to generate classifications or quantitative estimates, using structured input features derived from standardized analytical procedures [20]. The large volume of numerical data generated in laboratory testing provides a suitable basis for developing such models. Within hematological testing, CBC parameters represent a well-standardized, numerically structured set of measurements, making them appropriate for multivariable modeling. Tepakhan and colleagues applied combined decision-tree models to RBC indices to differentiate iron deficiency anemia from thalassemia, providing an example of computational models developed exclusively from laboratory variables [21].

In the present study, we evaluated whether CBC parameters could predict low ferritin and low transferrin saturation in a selected outpatient testing population. Two parallel supervised classification pipelines were developed for identifying low ferritin concentration and low transferrin saturation using exclusively hematological variables as input features. Among the evaluated classifiers, XGBoost achieved the highest AUC for both targets and remained significantly superior in direct ROC comparisons using the DeLong test. For the ferritin-based model, the positive predictive value was 0.79, indicating that a substantial proportion of subjects classified as positive had reduced ferritin concentrations. Conversely, the transferrin saturation model yielded a negative predictive value of 0.83, supporting its ability to reliably exclude reduced transferrin saturation. Considered together, these performance characteristics suggest that the two models may provide complementary information when interpreted within the same laboratory framework, one contributing to rule-in performance and the other to rule-out capability.

The consistently higher discriminative performance of tree-based boosting methods, particularly for the ferritin target, indicates that the association between erythrocyte indices and iron stores is likely characterized by nonlinear interactions that are not fully captured by linear approaches. This interpretation is further supported by the feature importance analysis. For the ferritin model, MCH emerged as the most influential predictor. This finding is biologically coherent and supported by previous evidence demonstrating a significant association between serum ferritin and MCH levels in patients with iron-deficiency anemia [22]. Reduced iron availability directly limits HB synthesis, leading to a decrease in HB content per erythrocyte before more pronounced changes in cell size occur. In this context, MCH may reflect iron-restricted erythropoiesis at an earlier or more quantitatively sensitive stage than other RBC indices. For the transferrin saturation model, MCV ranked highest in importance. This observation is consistent with the progressive reduction in erythrocyte size observed in iron-deficient states, in which sustained impairment of HB synthesis ultimately leads to microcytosis. The differential ranking of MCH and MCV across the two targets suggests that distinct hematological expressions of iron deficiency may be preferentially captured depending on whether the biochemical reference marker reflects iron stores or circulating iron availability.

Our findings are also in line with those reported by Garduno-Rapp and colleagues, who developed deep learning models for early identification of iron-deficiency anemia using 52 longitudinal laboratory parameters and achieved an AUROC of 0.89 with a gated recurrent unit architecture [23]. Although their approach incorporated a substantially broader panel of laboratory variables and a time-series structure, the overall discriminative performance observed in our study was comparable, despite using only age, sex, and routine CBC indices. This comparison suggests that a limited but biologically coherent hematological dataset may contain information associated with low ferritin and low transferrin saturation, even without extended biochemical panels or longitudinal data. Because the models are based only on age, sex, and routine CBC indices, they may be suitable for further evaluation in different laboratory settings. Their use in laboratories with limited access to iron studies, or as part of Laboratory Information System (LIS) workflows, cannot be inferred from the present pilot study. External validation, prospective assessment, and implementation studies are required before considering these applications in routine practice. At this stage, the models should be interpreted as tools for identifying hematological patterns that may prompt targeted biochemical assessment, not as substitutes for iron studies.

Recent studies have applied gradient-boosted decision tree models to routinely collected clinical and laboratory variables to identify iron deficiency in subjects without overt anemia [24]. These observations are concordant with the present findings and indicate that biochemical iron depletion may already be reflected in quantitative alterations of hematological indices before HB levels decline below the diagnostic threshold for anemia. The ability to capture these early changes through multivariable analysis supports the biological plausibility of the approach adopted in this study.

In the present analysis, hyperparameter tuning was explicitly performed to maximize AUC as the primary optimization criterion. Future studies may reasonably explore alternative tuning strategies tailored to specific laboratory applications, for example, prioritizing sensitivity in screening-oriented settings or specificity in confirmatory contexts. The results of the present analysis must be interpreted in light of the specific methodological framework in which the models were developed. Although performance was assessed on a held-out test subset, all data originated from a single institutional setting. As previously discussed, an independent validation on external cohorts, ideally generated on different analytical platforms and reflecting heterogeneous patient populations, may be advisable to determine the stability and broader applicability of the proposed models. It is also essential to clarify that the analytical strategy described herein is not conceived as a replacement for established diagnostic pathways. Iron deficiency remains a condition requiring biochemical confirmation and clinical contextualization. The potential contribution of a model based exclusively on CBC parameters lies in its ability to highlight hematological patterns that may warrant targeted second-line investigations, thereby supporting a more focused use of iron studies in routine laboratory practice. Although a post hoc power analysis showed high statistical power, the absence of an external validation cohort prevents assuming generalizability of the present findings to other clinical settings. Therefore, prospective validation on independent datasets remains necessary before clinical implementation. The study cohort was selected by design, since the inclusion required paired CBC and iron metabolism testing from the same blood collection; as a result, it may include a higher proportion of subjects investigated for suspected iron disorders, anemia, chronic disease, inflammatory conditions, or follow-up assessment. Performance may therefore differ in unselected CBC populations. The broad age range, together with the absence of clinical data and inflammatory markers, may have influenced target classification and model generalizability. Further studies should include external cohorts, age-stratified analyses, inflammatory markers such as C-reactive protein, and should be prospectively validated before considering screening or reflex-testing applications.

## 5. Conclusions

The present study provides a basis for the development of laboratory-based diagnostic support strategies for identifying low ferritin and low transferrin saturation from routine CBC parameters. This analysis is a widely available, standardized, and low-cost test routinely performed across different clinical settings. The observation that hematological parameters alone provide meaningful discriminatory performance suggests that information derived from routine hematological testing may assist in recognizing patterns associated with altered iron status, including contexts in which iron studies are not included in the initial test panel, are selectively requested, or are unavailable at the time of evaluation. This may also be relevant in low-resource settings, where a single routine analysis may provide an initial interpretation of the hematological profile and help identify patients who may require further evaluation of iron status. In this context, the CBC may help guide the use of dedicated iron studies and enhance the interpretive value of routine laboratory testing. Future integration of this approach into laboratory information systems may facilitate its use in routine clinical practice.

## Figures and Tables

**Table 1:** Results of the different classifiers on the holdout test for the low ferritin target.

Model	Accuracy	Balanced Accuracy	Sensitivity	Specificity	PPV	NPV	F1-Score	AUC	AUC 95% CI	Brier Score
Logistic Regression	0.634	0.620	0.458	0.781	0.624	0.645	0.528	0.663	0.652–0.674	0.228
Basic Decision Tree	0.768	0.766	0.746	0.786	0.734	0.796	0.740	0.811	0.802–0.819	0.171
Optimized Decision Tree	0.795	0.788	0.730	0.846	0.790	0.798	0.759	0.835	0.827–0.8440	0.156
Basic Random Forest	0.790	0.785	0.740	0.830	0.775	0.801	0.757	0.850	0.842–0.858	0.153
Tuned Random Forest	0.794	0.789	0.748	0.831	0.778	0.806	0.763	0.853	0.845–0.861	0.151
XGBoost	0.802	0.796	0.749	0.843	0.791	0.809	0.770	0.858	0.851–0.866	0.147
SVM	0.669	0.651	0.501	0.802	0.667	0.669	0.572	0.712	0.702–0.722	0.213
k-NN (tuned as k = 25)	0.656	0.643	0.525	0.760	0.635	0.669	0.575	0.696	0.685–0.706	0.218
Naive Bayes	0.640	0.624	0.482	0.766	0.620	0.651	0.543	0.665	0.654–0.676	0.237

AUC, Area under the curve; PPV, positive predictive value; NPV, negative predictive value.

**Table 2:** Results of the different classifiers on the holdout test for the low transferrin saturation target.

Model	Accuracy	Balanced Accuracy	Sensitivity	Specificity	PPV	NPV	F1-Score	AUC	AUC 95% CI	Brier Score
Logistic Regression	0.781	0.590	0.221	0.958	0.627	0.796	0.327	0.750	0.7383–0.7610	0.154
Basic Decision Tree	0.776	0.589	0.229	0.949	0.588	0.796	0.330	0.676	0.6644–0.6874	0.162
Optimized Decision Tree	0.798	0.652	0.371	0.933	0.636	0.824	0.468	0.742	0.7299–0.7539	0.151
Basic Random Forest	0.802	0.659	0.385	0.933	0.646	0.828	0.482	0.777	0.7663–0.7882	0.145
Tuned Random Forest	0.800	0.647	0.353	0.941	0.653	0.821	0.458	0.780	0.7692–0.7909	0.144
XGBoost	0.806	0.665	0.393	0.937	0.663	0.830	0.494	0.801	0.7911–0.8115	0.139
SVM	0.795	0.609	0.252	0.966	0.702	0.803	0.370	0.728	0.7150–0.7406	0.155
k-NN (tuned as k = 25)	0.794	0.631	0.317	0.944	0.643	0.814	0.425	0.768	0.7574–0.7795	0.148
Naive Bayes	0.7503	0.620	0.369	0.871	0.474	0.814	0.415	0.727	0.7151–0.7382	0.193

AUC, Area under the curve; PPV, positive predictive value; NPV, negative predictive value.

## Data Availability

The data presented in this study are available on request from the corresponding author.

## Abbreviations

The following abbreviations are used in this manuscript:

AUC	Area Under the Curve
CBC	Complete Blood Cell Count
CI	Confidence interval
F1-score	Harmonic mean of precision and sensitivity
HB	Hemoglobin
HT	Hematocrit
K-NN	k-nearest neighbors
LIS	Laboratory Information System
MCH	Mean Corpuscular Hemoglobin
MCHC	Mean Corpuscular Hemoglobin Concentration
MCV	Mean Corpuscular Volume
NPV	Negative Predictive Value
PPV	Positive Predictive Value
RBC	Red Blood Cell
RDW	Red blood cell distribution width
ROC	Receiver Operating Characteristics
SVM	Support vector machine
TSAT	Transferrin saturation
XGBoost	Extreme gradient boosting
